# Current Omics Trends in Personalised Head and Neck Cancer Chemoradiotherapy

**DOI:** 10.3390/jpm11111094

**Published:** 2021-10-26

**Authors:** Loredana G. Marcu, David C. Marcu

**Affiliations:** 1 Faculty of Informatics & Science, University of Oradea, 410087 Oradea, Romania; 2 Cancer Research Institute, University of South Australia, Adelaide, SA 5001, Australia; 3 Faculty of Electrical Engineering & Information Technology, University of Oradea, 410087 Oradea, Romania; david@marcunet.com

**Keywords:** squamous cell carcinoma, toxicity, drug resistance, radiomics, dosiomics, pharmacomics

## Abstract

Chemoradiotherapy remains the most common management of locally advanced head and neck cancer. While both treatment components have greatly developed over the years, the quality of life and long-term survival of patients undergoing treatment for head and neck malignancies are still poor. Research in head and neck oncology is equally focused on the improvement of tumour response to treatment and on the limitation of normal tissue toxicity. In this regard, personalised therapy through a multi-omics approach targeting patient management from diagnosis to treatment shows promising results. The aim of this paper is to discuss the latest results regarding the personalised approach to chemoradiotherapy of head and neck cancer by gathering the findings of the newest omics, involving radiotherapy (dosiomics), chemotherapy (pharmacomics), and medical imaging for treatment monitoring (radiomics). The incorporation of these omics into head and neck cancer management offers multiple viewpoints to treatment that represent the foundation of personalised therapy.

## 1. Introduction

The heterogenous nature of head and neck carcinomas (HNCs) renders this malignancy challenging to cure. While in an early stage resectable cancers are usually treated with single-modality treatment in the form of surgery or radiotherapy with very good disease control and survival, locally advanced head and neck carcinomas are managed with combined therapies that usually include surgery followed by risk-adapted chemo/radiotherapy or primary chemotherapy alone. Nevertheless, the long-term prognosis for advanced HPV-negative HNC has not improved significantly over the last few decades, the overall survival being often burdened by long-term toxicities [1].

Next to tumour staging, biological factors such as tumour repopulation, acute and chronic hypoxia, cancer stem cell density, DNA damage repair and, more generally, a high degree of tumour heterogeneity have a strong influence on tumour response to treatment [2]. Tumour markers related to the above factors were shown to have independent prognostic value in advanced HNC patients, illustrating the important and individual role of biological factors for locoregional control in these patients [2]. In-depth patient-specific evaluation of biological parameters and their impact on treatment outcome is the key to personalised therapy.

Precision medicine is a commonly encountered terminology which focuses on medicine that is tailored to a subgroup of patients based on similar genetic/epigenetic features or medical-imaging-based diagnostic characteristics. This stratification of patients is usually met within current clinical trials. As opposed to precision medicine, personalised therapy aims to target the individual patient rather than a subgroup, offering a more customised treatment.

Lately, research has focused on correlating validated imaging biomarkers for specific biological parameters (hypoxia, proliferation, stemness, etc.) with ‘omics’ features, using machine learning algorithms in order to achieve better patient stratification.

The aim of this work is to present the newest trends in personalised chemoradiotherapy of head and neck cancer by discussing the findings of the latest omics involving radiotherapy (dosiomics), chemotherapy (pharmacomics), and medical imaging for treatment monitoring (radiomics). It is postulated that by incorporating these omics into the management of head and neck cancer, a holistic approach to treatment can be achieved which represents the next step in personalised therapy.

## 2. Radiomics in Head and Neck Cancer

Recent employment of artificial intelligence in image processing has shown the potential for the identification of highly detailed and robust tumour imaging features that are not perceptible by the naked eye, known as radiomic features. This information is used for the development of radiomics-based machine learning models for clinical decision support [3].

Radiomics in head and neck cancer research is employed for several purposes, including automated radiation targeting using region-based PET/CT texture analysis [4], automatic detection and classification of head and neck cancer subtypes based on PET/CT support vector machines [5], outcome prediction in terms of tumour control (Table 1) [6,7,8], and outcome prediction of normal tissue toxicity [9,10,11,12,13,14].

Given the radiobiological challenges encountered during HNC treatment, radiomics has found a way to be utilised for the identification of treatment-resistant sub-volumes that are caused by various factors, such as hypoxia, a high proliferation rate, inherent resistance, or the existence of cancer stem cells [15]. Differences in radiomics features extracted from various tumour sub-volumes allow for a better characterisation of the targeted region, information that serves treatment adaptation and potential dose intensification in the areas that show resistance to therapy. As stipulated by Aerts et al., a prognostic radiomics signature that depicts tumour heterogeneity is correlated with gene expression patterns [16]. Quantification of medical image features combined with individual genomic phenotypes reveals radiogenomic characteristics of a tumour, facilitating decision making in both diagnosis and treatment.

The human papilloma virus (HPV) is another key factor that differentiates among HNC regarding patient response to treatment. The clinical evidence where HPV-positive HNC patients (oropharyngeal, HPV/p16) show better outcomes to conventional therapy than their HPV-negative counterparts [17] was further investigated by researchers for the potential role of radiomics in identifying radiological differences between the two tumour subtypes. Cantrell et al. reported on a blinded matched-pair analysis based on pretreatment CT images aiming to evaluate pattern differences among patients as a function of HPV status. The study showed that HPV-positive tumours have well-defined boundaries, while HPV-negative tumours present with poorly defined margins that are prone to invade adjacent tissues [18]. Using contrast-enhanced CT images from patients with oropharyngeal carcinoma and known HPV status, Buch et al. reported statistically significant differences in a number of radiomic features as a function of HPV: histogram feature median (*p* = 0.006), histogram feature entropy (*p* = 0.016), and grey-level co-occurrence matrix features (*p* = 0.043) [19]. Within a study incorporating non-oropharyngeal cancer patients with known HPV status, Fujita et al. confirmed previous findings that contrast-enhanced CT for initial staging allowed the identification of texture features that distinguish between tumours as a function of HPV status [20]. Based on image feature analysis that encompassed five histogram features (*p* ≤ 0.03), three grey-level co-occurrence matrix features (*p* ≤ 0.02), one grey-level run length feature (*p* = 0.009), two grey-level gradient matrix features (*p* ≤ 0.02), and five Law features (*p* ≤ 0.04), the study demonstrated that morphologic differences characteristic to HPV status can be identified in non-oropharyngeal cancers as well, thus proving the utility of radiomics as an HPV classification tool.

A large number of studies have demonstrated the usefulness of radiomics in terms of normal tissue toxicity prediction after radiotherapy to allow for adaptive therapy planning in patients at high risk of developing acute and late side effects [9,10,11,12,13,14]. A special focus is on the association between the post-radiotherapy structural and textural changes in the parotid gland and the level of xerostomia. It is known that radiation-induced parotid shrinkage leads to a shift of the gland towards the head midline that is generally the high-dose region; thus, the parotid will be affected by a larger dose than planned. Most radiomics studies conclude that conventional imaging techniques used in HNC (CT, MR, and ultrasound) offer a set of textural features that can serve as predictors for xerostomia, thus identifying the subgroup of patients that requires the adaptation of treatment planning to reduce toxicity (see Table 1). Beside xerostomia, machine learning was employed to identify radiomics features that correlate with a high risk of radiation-induced hearing loss [21] and whole-brain white matter injury after radiotherapy [22].

**Table 1:** Radiomic studies in HNC, studying tumour control and normal tissue toxicity.

Study Type/Goal	Imaging Modality/Radiomic Features	Observations/Conclusions
**Radiomic Studies for Outcome Prediction: Tumour Control**		
Staging and risk stratification model in oropharyngeal carcinomas (Cheng et al., 2013) [23]	*PET/CT imaging* Grey-level co-occurrence matrix, uniformity, and coherence	A risk stratification strategy was developed based on total lesion glycolysis (TLG) and uniformity. TLG, uniformity, and HPV positivity are significantly associated with overall survival.
Prognostic imaging biomarkers for overall survival (Parmar et al., 2015) [24]	*CT imaging* First-order intensity statistics, shape, and volume textural featuresArea under receiver operating characteristics curve (AUC) used to quantify the prognostic performance of different feature selection	Three feature selection methods—minimum redundancy maximum relevance, mutual information feature selection, and conditional infomax feature extraction had high prognostic stability and performance for the prediction of overall survival.
Prediction of local control using pre-treatment PET vs. CT (Bogowicz et al., 2017) [6]	*PET/CT imaging* CT density, HLH intensity, grey-level size zone texture matrices, and spherical disproportion	The model overestimated tumour control probability in high-risk patients. Combined PET/CT added no extra value when compared to either imaging method alone.
Risk prediction models of locoregional recurrences and distant metastases (Vallieres et al., 2017) [7]	*CT imaging* Large zone high grey-level emphasis; zone size non-uniformity	No significant correlation was found between radiomic features and locoregional recurrence. Image-derived features combined with clinical variables offer the highest predictive values.
Risk prediction models of all-cause mortality, local failure & distant metastasis (Folkert et al., 2017) [8]	*CT imaging* Image features: statistical, shape, and texture	Multiparametric models had the strongest predictive power. The local failure model demonstrated robustness when conveyed onto independent patient cohorts.
**Radiomic Studies for Outcome Prediction: Normal Tissue Toxicity**		
Assessment of structural changes in parotid glands (Scalco et al., 2013) [9]	*CT imaging* Textural features and gland volume	Variations in mean intensity and fractal dimension (after the second and last week of radiotherapy) were the best predictors of parotid shrinkage.
Early prediction of parotid shrinkage and toxicity (Pota et al., 2017) [10]	*CT imaging* Textural features, spatial patterns, fractal dimensions, and gland volume combined with fuzzy classification	The final parotid shrinkage rate strongly correlated with 12-month xerostomia: glands that presented strong volume variation post-radiotherapy could be less affected by late xerostomia.
Prediction of radiation-induced xerostomia and sticky saliva (Van Dijk et al., 2017) [11]	*CT imaging* Geometric features, CT intensity, and textural features	Prediction of 12-month xerostomia and sticky saliva were improved by the addition to the initial CT image biomarkers of the short-run emphasis (quantifies heterogeneity of parotid) and of the maximum CT intensity of the submandibular gland (gland density).
Prediction of late xerostomia using parotid gland fat (Van Dijk et al., 2018) [12]	*MR imaging* T1-weighted MR-image-based intensity (90th intensity percentile) and textural features	The ratio of fat-to-functional-parotid-tissue is associated with 12-month xerostomia. MR-based radiomics improved prediction.
Prediction of severe late xerostomia (Nardone et al., 2018) [25]	*CT imaging* Textural features: grey-level co-occurrence matrix (GLCM), neighbourhood grey-level dependence matrix (NGLDM), grey-level run length matrix (GLRLM), grey-level zone length matrix (GLZLM), sphericity, indices from the grey-level histogram, and parotid volume	Parameters with the strongest correlation with severe chronic xerostomia: V30, mean dose, kurtosis, grey-level co-occurrence matrix (GLCM), and run length non-uniformity (RLNU). CT texture analysis could allow for the enhancement of dose constraints to organs at risk to avoid severe side effects.
Multivariable modelling study of chemotherapy-induced hearing loss (Abdollahi et al., 2018) [21]	*CT imaging* Textural features with highest predictive power: intensity histogram (IH) and grey-level co-occurrence matrix (GLCM)	Ten machine learning classifiers used for radiomic feature selection, classification, and prediction, with over 70% accuracy. No single algorithm showed superiority for all problems. CT image features of cochlea can serve as biomarkers for predicting hearing loss after therapy.
Early prediction of acute xerostomia during therapy (Wu et al., 2018) [26]	*CT imaging* Histogram-based features: mean CT number (MCTN), volume, skewness, kurtosis, and entropy	Daily CT images analysis during IMRT indicated that changes in gland volume or MCTN are not correlated with grade of xerostomia if considered separately but when combined in a CT-based xerostomia score model. This can predict severity with a precision of 100% at the 5th week of therapy.
Assessment of whole-brain white matter injury after radiotherapy (Leng et al., 2019) [22]	*MR diffusion tensor imaging* Fractional anisotropy (FA) and FA skeleton matrix	Post-therapy decreased FA values (that quantify the degree of water diffusion in cerebral white matter) showed microstructure damage of the white matter. Radiation brain injury in HNC patients can be quantified using MR diffusion-tensor-based radiomics.
Predictive model of acute radiation-induced xerostomia (Sheikh et al., 2019) [13]	*CT and MR imaging* Shape, first-order statistics, grey-level co-occurrence matrix (GLCM), grey-level run length matrix (GLRLM), and grey-level size zone matrix (GLSZM)Model performance improved when DVH was combined with CT and MRI features	Higher-order texture features for salivary glands were key predictors of xerostomia. MRI: patients with xerostomia appear more heterogeneous and hypointense. CT: submandibular glands of patient with xerostomia appear more hypodense and heterogeneous. Baseline CT and MRI features can potentially reflect baseline salivary gland function and the risk of radiation-induced effects.
Longitudinal study on post-radiotherapy parotid gland changes in nasopharyngeal cancer patients (Wu et al., 2020) [14]	*MR and ultrasound (US) imaging* MRI features: volumeUS features: echogenicity and hemodynamic parameters (resistive index, pulsatility index, and peak diastolic and end-diastolic velocity)	Parotid and submandibular gland shrinkage associated with xerostomia was observed post-radiotherapy (IMRT), with most significant changes detected after 6 months. Mild correlation found between gland dose and post-radiotherapy gland volume.

Lately, there is research interest in combining clinical, dosimetric, and radiomic features in classification models to increase their predictive power for both tumour response and normal tissue toxicity [10]. This approach offers a more comprehensive understanding of the patient’s individual characteristics, allowing for tailored management from diagnosis to treatment follow-up.

## 3. Dosiomics in Head and Neck Cancer

Radiotherapy is the main therapeutic choice for the locoregional management of unresectable head and neck carcinomas. To optimise radiation delivery to the target volume, treatment techniques have evolved from 2D to 3D conformal radiotherapy, then further to intensity-modulated radiation therapy (IMRT) using fixed beam angles. More recently, rotational IMRT such as VMAT/RapidArc is employed to create conformal dose distribution via rotational techniques with dose intensity modulation. The steep dose gradient achieved with VMAT offers better normal tissue sparing, usually with fewer monitor units than IMRT and shorter treatment delivery, thus also increasing patients’ comfort [27].

Treatment planning verifications concerning both dose prescription to the target volume and dose constraints for the organs at risk involve dose-volume histograms (DVH) that provide quantitative analysis of the dose delivered to different volumes, though without supplying spatial information. This aspect is a clear shortcoming of the treatment plan evaluation, as the lack of spatial dosimetric data can lead to omissions within the target and/or overdose of critical normal structures. Both consequences can result in unwanted outcomes that are difficult or even impossible to rectify. For instance, while clinical studies established that the mean dose to the parotid gland is a good predictor of xerostomia, this measure failed to identify patients at risk in larger cohorts where most patients met the dose constraints, leading to adverse effects [28,29].

Since accurate prediction of normal tissue toxicity based on DVH is not achievable, a number of studies have embraced machine learning classifiers in normal tissue complication probability (NTCP) models to identify dosimetric or patient-related factors that could predict severe side effects such as xerostomia. Using data from the PARSPORT phase III trial (parotid-sparing IMRT in patients with HNC), Buettner et al. have developed dose–response models by using Bayesian logistic regression and spatial dose distribution (dose-shape features) in the submandibular gland while also including clinical and radiobiological factors (such as regional variations in radiosensitivity of the parotid glands) for outcome prediction [30]. By analysing the spatial dose distribution pattern of the parotid, it was observed that xerostomia can be limited or even avoided by minimising the dose to the lateral and cranial sections of the gland. Moreover, data analysis showed that resection of the submandibular gland considerably increased the risk of this side effect. The study concluded that models that take into account the shape of the dose distribution are significantly better predictors of xerostomia than standard mean-dose models.

The use of machine learning techniques for the analysis of spatial dose distribution in radiotherapy was coined dosiomics. Considered as an extension of radiomics, dosiomics entails the extraction of spatial features of the dose distribution within the investigated organ, features that are further used to build prediction models with machine learning classification algorithms.

In both disease control and normal tissue toxicity prediction models, dosiomics is often integrated with radiomic features as well as other clinical and/or patient-related factors, to increase the predictive power of omics-based models. In view of this, Gabryś et al. designed a study to evaluate the precision of a radiomics–dosiomics model to predict the risk of xerostomia among HNC patients when compared to the more classical NTCP models that are based on the mean dose to the parotid [31]. The modelling study was built on a cohort of 153 HNC patients’ data and aimed to evaluate early, late-, and long-term xerostomia after radiotherapy. Radiomic features included the shape of the parotid (volume, area, sphericity, compactness, and eccentricity) while dosiomic features encompassed dose shape (DVH, spatial dose gradient, spread, correlation, and skewness). Univariate analysis showed that parotid and dose-shape features are very good predictors of xerostomia, the highest risk group in developing long-term effects consisting of patients with a small parotid gland (9.55 mm^3^ median volume high-risk vs. 14.37 mm^3^ low-risk) and steep dose gradients in the right–left direction (1.7 Gy/mm high-risk vs. 1.2 Gy/mm low-risk). Multivariate analysis highlighted the important role of personalised treatment planning, as the models showed strong patient specificity. Thus, females with small and elongated parotid presented with a higher risk of long-term xerostomia compared to males with large and round glands. The most representative dosiomic feature was the spread of the contralateral DVH that quantifies the standard deviation of the parotid dose. The authors concluded that in a highly conformal radiotherapy regimen, dosiomic features (dose shape) add value to treatment outcome prediction modelling, which combined with patient-specific factors (parotid shape, sex) lead to a more accurate personalised risk assessment [31].

In a recent study conducted on 237 patients with HNC, Wu et al. investigated the predictive power of radiomics and dosiomics for locoregional recurrence after IMRT [32]. Radiomics features were extracted from both CT and PET scans and selected according to their concordance index values, which were condensed via principal component analysis. The condensed features served as input parameters for multivariate Cox proportional hazard regression models. The dosiomics prognostic model, which was built on similar initial features, additionally included 3D dose distribution data from IMRT treatment plans. Results showed that while the integration of dosiomics into radiomics lead to a successful patient classification into different risk groups, the radiomics-only model was not able to offer an accurate stratification, thus highlighting the importance of volumetric knowledge of dose distribution for outcome prediction after radiotherapy [32].

While dosiomic research is still in its early phases of development and application, studies so far showed that the inclusion of 3D dosimetric texture analysis in predictive modelling can improve prediction accuracy, highlighting the need to surpass current DVH constraints in radiotherapy planning for a more personalised approach.

## 4. Pharmacogenomics and Pharmacogenetics in Head and Neck Cancer

### 4.1. Introduction—Chemotherapy in Head and Neck Cancer

There is clear evidence through randomised clinical trials that the addition of chemotherapy to radiation improves both locoregional control and survival among HNC patients compared to radiotherapy alone [33]. While there are continuous advances in targeted and immune-therapy, combined chemoradiotherapy is still the standard of care for locally advanced head and neck cancer, with cisplatin-based chemotherapy as the mainstay of first-line treatment.

One of the main shortcomings of cisplatin is the drug-induced normal tissue toxicity that is often a dose-limiting factor. Another limitation of cisplatin administration is tumour resistance to the drug, which is often developed during therapy, drastically decreasing its efficacy [34,35]. Cisplatin is rarely administered as a sole chemotherapy agent and is most often combined with different classes of drugs to offer better radiosensitisation of tumour cells and to overcome drug resistance. Beside alkylating agents, other commonly used drug classes in HNC management are antimetabolites, antibiotics, topoisomerase inhibitors, and taxanes. More recently, targeted agents in the form of monoclonal antibodies have been used in combination with conventional chemotherapy, agents that were designed to target programmed cell death protein 1 (PD-1), which is often overexpressed in tumour cells, or the epidermal growth factor receptor (EGFR), which is also overexpressed in 90% of HNC patients and is associated with poor outcomes [36].

Owing to the large tumour heterogeneities among head and neck malignancies and considerable inter-patient variation regarding tumour response and adverse effects to chemoradiotherapy it is greatly challenging, if at all possible, to define the optimal chemotherapy cocktail for HNC patients. Furthermore, considering the current trends in dose de-escalation and the limitation of chemotherapy to avoid unnecessary toxicities among well-responding non-smoking HNC patient subgroups with HPV-positive cancers, a more personalised approach would allow better patient stratification without compromising the expected therapeutic outcome [37,38]. This aspect of treatment optimisation has stimulated new research avenues into personalised therapy through pharmacogenomics and pharmacogenetics to establish correlations between patients’ genetics characteristics and response to specific drugs.

### 4.2. Pharmacomics—Towards Personalised Chemotherapy

Pharmacomics is a new research field that encompasses pharmacogenomics and pharmacogenetics, aiming to investigate the genetic basis of individualised responses to various drugs. In cancer research, pharmacomics shows promise in new drug development as well as in better management of cancer patients in need of chemotherapy. Given that drug-caused toxicity is often added to radiation-induced normal tissue effects, it is critical for cancer patients to receive chemotherapeutic agents that are more compatible with their genetic makeup in order to maximise tumour response and to minimise normal tissue toxicity. Furthermore, knowing the genetic basis for drug resistance in individual patients would point towards the administration of more compatible agents. In view of the above, pharmacomics could play an important role in personalised chemotherapy.

Tumour heterogeneity and the high genomic instability identified in the genomes of malignant cells lead to treatment resistance, tumour progression, and an overall aggressive nature in tumours. The latest developments in genome research have resulted in advancements from microarray-based platforms to next-generation sequencing (NGS) technologies, with more user-friendly software for data analysis and reduced costs for data storage. NGS-based research has focused on the identification of molecular mechanisms behind carcinogenesis, tumour progression, and distant spreading of cancer cells, while uncovering new oncogenes, tumour suppressor genes, and tumour-specific signalling pathways.

In head and neck cancer, the most frequently mutated genes known from earlier genomic research include TP53 (with up to 67.5% frequency of mutations), CDKN2A, and PIK3CA (around 16.5% mutated) [39]. NGS analysis identified additional genes that are commonly mutated in HNC, revealing NOTCH1 as the third most frequent mutation, observed in up to 15% of the studied HNC cell lines [39].

Beside mutations in the TP53 gene, recurrent mutations in caspase-8 (CASP8), which initiates programmed cell death, were also found with high frequency in HNC. It was shown that the expression of CASP8 triggers apoptosis caused by chemotherapeutic agents through the production of reactive oxygen species inside the cell [40]. Agents such as taxanes were observed to promote CASP8-mediated programmed cell death, whereas mutations in this gene could lead to drug resistance.

Mutations in a number of other genes found in HNC were also shown to be responsible for resistance to chemotherapy. Yamano et al. have identified five genes, LUM, PDE3B, PDGF-C, NRG1, and PKD2, that showed a strong correlation with cisplatin resistance in HNC cell lines that could serve as predictors of treatment efficacy [41]. Overexpression of the cell surface transmembrane glycoprotein CD147 is widely found in a variety of cancers, including head and neck, and is responsible for cancer progression, metastases, and chemoresistance. Studies investigating the mechanisms of resistance to chemotherapy revealed that CD147 was overexpressed in cisplatin-resistant HNC, causing not only cisplatin but multidrug resistance. Downregulation of CD147 expression by deactivating the MAPK/ERK signalling pathway led to increased sensitivity to the drug, which could represent a therapeutic target for the affected tumours [42,43].

A common drug cocktail employed in HNC chemotherapy due to its potential synergistic effect is the combination of cisplatin, docetaxel, and 5-FU. To elucidate some of the mechanisms behind drug resistance triggered by this drug combination, triple-drug-resistant cell lines were generated by exposing the cell lines to increasing drug concentrations [44]. The study aimed to evaluate both cellular and molecular effects by assessing cell viability, cell cycle properties, apoptosis, and gene expression associated with multidrug resistance. The mRNA expression levels of the following genes associated with multidrug resistance were determined: MDR1, MRP2, ERCC1, CTR1, survivin, and thymidylate synthase (TS). On the cellular level, the resistant cell lines showed prolonged arrest in the G2/M phase, a common mechanism exhibited by cancer stem cells to evade apoptosis. On the molecular level, overexpression of the ERCC1 and upregulation of CTR1 gene were observed, both mutations being indicative of cisplatin resistance: platinum-based drugs are predominantly linked to and eradicate cancer cells with negative ERCC1 expression, and the expression of the copper transporter receptor 1 (CTR1) is correlated with intratumoral platinum accumulation, with increased expression being indicative of poor uptake. Overexpression of the MRP2 gene was shown to mediate docetaxel resistance in cisplatin-resistant cell lines, thus suggesting a role in multidrug resistance. An increased expression of thymidylate synthase, also observed in this study, is a mechanism that underlies resistance to 5-FU. Overall, expression profiling analysis revealed a synergistic effect of multidrug resistance genes, suggesting that sequential drug administration could lead to better sensitisation compared to combined treatment [45]. For instance, in a study on oral cancers, Tamatani et al. observed that the antitumour efficacy of 5-FU is enhanced by prior administration of docetaxel, thus deeming sequential docetaxel followed by 5-FU a more efficient treatment strategy in HNC than combined chemotherapy [45]. Table 2 is a compilation of gene mutations responsible for chemotherapy resistance.

Next to drug resistance, chemotoxicity is another factor limiting the success of chemotherapy. The results of a preliminary study on genetic variability and chemotoxicity after the administration of 5-FU and cisplatin found a significant correlation between the variants of glutathione S-transferase Mu 1 (GSTM1) and cisplatin toxicity (*p* = 0.043) [48]. No association between 5-FU-toxicity-related genes (DPYD, TYMP, and MTHFR) and grade 3/4 toxicity was observed, though this might be due to the small number of patients enrolled in the study (23 HNC patients). For the same reason, no combination of genetic variants responsible for normal tissue toxicity could be identified within the study, thus justifying investigations on larger sample sizes.

Research showed that 5-FU toxicity is primarily linked to deficiency in dihydropyrimidine dehydrogenase (DPYD), a key metabolic enzyme, which is due to a deleterious polymorphism in the gene that encodes DPYD. A meta-analysis of individual patient data encompassing 7365 cancer patients has confirmed the association between a number of variants of DPYD and severe toxicity (grade 3/4), including hematologic and gastrointestinal, in patients treated with fluoropyrimidines either as single agents or in combination with other drugs and/or radiotherapy [49]. The most relevant clinical predictors of 5-FU toxicity were identified in the form of two DPYD variants (c.1679T > G and c.1236G > A/HapB3), and pre-treatment screening for these variants is therefore recommended to find treatment alternatives and to reduce normal tissue toxicity.

In view of the above, a prospective, multicentre, drug safety analysis was conducted on 1103 cancer patients that were planning to commence a fluoropyrimidine-based chemotherapy regimen [50]. The results showed higher rates of severe toxicity in DPYD variant carriers than in wild-type patients (*p* = 0.0013), indicative of dose reduction requirements for patient safety. Thus, for patients with DPYD*2A and c.1679T > G carriers, a 50% dose reduction was suggested to limit severe adverse effects. The findings of the study support the need of genotype-guided personalised drug dosing as a new standard of care in chemotherapy [50].

The large number of genes and their variants that are being identified to be responsible for drug resistance and toxicity in head and neck cancer represent a pool of valuable data for big data analysis. These data require accurate interpretation to find correlations among the genetic variants and the pursued outcome in order to help identify those patient groups that are at high risk of developing chemotherapy resistance or severe side effects. Studies found that differences in sex, ethnicity, and chemotherapy regimen are additional factors that influence the heterogeneity of genetic variants connected to drug toxicity [51]; therefore, the use of machine learning in data processing and analysis could assist in handling large amounts of data and accelerate the implementation of genotype-guided chemotherapy.

Research into pharmacogenetics has been advancing at a fast pace in the last decade; however, clinical implementation of research results is lagging behind. A recent European survey of healthcare professionals found that although 84% of respondents have considered pharmacogenomics relevant to their practice, two-thirds had not ordered a pharmacogenomic test in the year prior to the survey [52]. Large-scale initiatives exist to encourage implementation of pharmacogenomics research results in clinical practice, such as the Clinical Pharmacogenetics Implementation Consortium (CPIC) in the USA and the Ubiquitous Pharmacogenomics program (U-PGx) in Europe [53].

Advances in this field are dependent on developments in artificial intelligence and the availability of public genome datasets coupled with large datasets of anonymised electronic health records.

## 5. Chemoradiotherapy Response Monitoring Using Delta-Radiomics

As shown in the radiomics section, imaging plays an integral part in treatment response monitoring of oncological patients. Post-therapy imaging assessment supplies information on the success of therapy. Yet, the analysis of images acquired during treatment has the potential to assist with treatment adaptation, thus offering a more personalised therapy. Recent studies of serial PET/CT images acquired throughout the treatment course have shown that the evaluation of tumour sub-volume dynamics can facilitate adaptive treatment approaches [54,55].

In their study on head and neck cancer patients, Lazzeroni et al. investigated the correlation between the dynamicity of hypoxia throughout radiochemotherapy assessed via sequential PET/CT imaging and outcome prediction. Hypoxia is associated with resistance to treatment and recurrence. The use of a hypoxia-specific PET radiotracer 18FMISO (18F-fluoromisonidazole) allowed for the generation of oxygen partial pressure maps to evaluate the evolution and severity of hypoxic sub-volumes within the target. Comparative longitudinal analysis of the oxygen partial pressure maps demonstrated correlations between the hypoxia sub-volumes and treatment outcomes, such as locoregional recurrence. Features derived from the first two weeks of treatment showed potential to predict outcomes in hypoxic head and neck cancer patients [54].

In a similar study, Sörensen et al. showed that textural features of hypoxia-specific FMISO-PET/CT images as well as changes in radiomics features during chemoradiotherapy predict survival in head and neck cancer. The study accrued 29 HNC patients that underwent FMISO-PET/CT for hypoxia evaluation before and during chemoradiotherapy. For all scans, the first-order metrics tumour-to-background ratio, coefficient of variation, total lesion uptake, and integral non-uniformity were calculated, together with three second-order textural features from grey-level matrices and the differential non-uniformity to show regional changes within the field of view. Prognostic groups were separated based on differential non-uniformity before and during chemoradiotherapy (week two) and non-uniformity from the grey-level run length matrix in week two. The study showed that textural features on FMISO-PET scans before and during chemoradiotherapy (week two) were good indicators of patient outcome, as tumours with higher homogeneity of hypoxia throughout the course of treatment correlated with a better response. The standard FDG-PET scan before treatment did not serve as an outcome predictor [55].

The principle of delta-radiomics was applied in most studies investigating the correlation between radiomics features at various time points (before, during, and after radiotherapy/chemoradiotherapy) and the severity of treatment-induced side effects (Table 1). Most investigations presented in Table 1 under radiomic studies for outcome prediction in the context of normal tissue toxicity have determined textural feature variations and volume changes in the parotid gland to assess the role of these variations in predicting severe xerostomia [9,10,14,26].

## 6. Conclusions

Head and neck cancers present an exclusive set of diagnostic and therapeutic challenges owing to their complex radiobiological behaviour and tumour heterogeneity. Despite all efforts to improve treatment delivery techniques and to administer more targeted therapies to limit normal tissue toxicity, the long-term prognosis in head and neck cancer patients is still limited.

Current implementations of the omics results are mainly focused on treatment outcome prediction and risk of recurrence rather than on direct personalisation of individual patient therapy [56]. It is expected that future developments in the discussed omics fields will influence the treatment of the individual patient from first contact with the healthcare system by allowing clinicians to deploy a truly individualised treatment based on radiomic analysis of diagnostic imaging, on detailed dosiomic analysis to target the tumour volume with utmost precision, alleviating side effects on healthy tissues, and on pharmacogenetic tests that enable the choice of the most effective drug family for the individual patient.

## Figures and Tables

**Table 2:** Gene mutations in HNC responsible for chemotherapy resistance.

Resistance to Chemotherapy Agent/Group of Agents	Gene Mutation Responsible for Chemoresistance	Normal Function of the Gene
Platinum compounds (cisplatin and carboplatin)	Copper transporter receptor 1 (CTR1) (Govindan et al., 2015) [44]	Major copper influx transporter in cells
	Excision repair cross-complementation group 1 (ERCC1) (Govindan et al., 2015) [44]	Critical role within the nucleotide excision repair system of DNA
	Excision repair cross-complementation group 4 XPF (ERCC4) (Vaezi et al., 2011) [46]	Protein involved in DNA binding and protein–protein interaction
	Lumican (LUM) (Yamano et al., 2010) [41]	Proteoglycan involved in epithelial cell migration and tissue repair
	Cyclic nucleotide phosphodiesterase type 3 (PDE3B) (Yamano et al., 2010) [41]	Intracellular messengers that regulate numerous signalling pathways
	Platelet-derived growth factor C (PDGF-C) (Yamano et al., 2010) [41]	Important role in connective tissue growth and function. Belongs to the PDGF/vascular endothelial growth factor family
	Neuregulin-1 (NRG1) (Yamano et al., 2010) [41]	A glycoprotein produced in a variety of isoforms that induce cell growth and differentiation
	Protein kinase D (PKD2) (Yamano et al., 2010) [41]	Propagates growth factor receptors at the cell surface
	Emmprin (CD147) (Huang et al., 2013, and Ma et al., 2017) [42,43]	Extracellular matrix metalloproteinase inducer
Antimetabolites (5-FU)	Thymidylate synthase (TS) (Govindan et al., 2015) [44]	Key enzyme in DNA biosynthesis
Taxanes (docetaxel and paclitaxel)	Caspase-8 (CASP8) (Stupack et al., 2013) [40]	Initiates programmed cell death
	Forkhead box protein C2 (FOXC2) (Zhou et al., 2015) [47]	Role in the development of mesenchymal tissues
